# First Aid to the Injured and Sick

**Published:** 1902-02

**Authors:** 


FIRST AID TO THE INJURED AND SICK. By F. J. WARWICK, B. A., M. B., Cantab., Associate of King's College, London; Surgeon-Captain, Volunteer Medical Staff Corps, London Companies, etc.; and A. C. TUNSTALL, M. D., F. R. C. S. Ed., Surgeon-Captain Commanding the East London Volunteer Brigade Bearer Company; Surgeon to the French Hospital and to the Children's Home Hospital, etc 16mo volume of 232 pages and nearly 200 illustrations. Philadelphia and London: W. B. Saunders & Co. 1901. [Price, cloth, $1.00 net.]

This book was written for the purpose of putting in the hands of the laity practical information as to rendering immediate temporary assistance to the sick or injured before the arrival of a doctor.

Part I. is a brief outline of the structure and functions of the human body. Part II. begins with the application of bandages. Then follow in order chapters on the immediate treatment of hemorrhage, wounds, dislocations and fractures; asphyxia, poisoning, insensibility, burns and scalds; transportation of the sick and injured, etc.

The instructions given for dealing with the various emergencies discussed are simple, practical and clearly stated. Those whose work renders them liable to have to deal with accidents, as railway employees, policemen, etc., ought to find the book one of great value.—M.J.F.

